# The safety and response of CT guided percutaneous cryoablation for lung nodules by 17-gauge needles

**DOI:** 10.1186/s12880-023-01110-6

**Published:** 2023-10-09

**Authors:** Wei-Chan Lin, Po-Ju Chen, Shelly Yim, Hsueh-Han Wang, Pen-An Liao, Chia-Yu Tai, Ming-Hong Yen

**Affiliations:** 1 School of Medicine, Fu-Jen Catholic University, New Taipei City, 24205 Taiwan; 2 Department of Radiology, Cathay General Hospital, No.280 Sec 4 Ren-Ai Rd, Taipei, 10630 Taiwan; 3 Department of Chest Surgery, Cathay General Hospital, Taipei City, 10630 Taiwan

**Keywords:** Cryoablation, Lung nodules, 17-Gauge Needles

## Abstract

**Background:**

The safety and efficacy of 17-gauge needles used in CT-guided percutaneous cryoablation for lung nodules were explored in this study. The purpose of the study was to compare the findings with earlier research and multi-center clinical trials that used various needle sizes.

**Methods:**

Between 2016 and 2020, a retrospective study was conducted with approval from the institutional review board. A total of 41 patients were enrolled, and 71 lung nodules were treated in 63 cryoablation procedures using local anesthesia. Complication rates were recorded, and overall survival rates as well as tumor progression-free rates were calculated using the Kaplan-Meier method.

**Results:**

Self-limited hemoptysis was caused by 12.9% of the procedures, and drainage was required for pneumothoraces resulting from 11.3% of them. The overall survival rates at one, two, three, and four years were 97%, 94%, 82%, and 67%, respectively. The tumor progression-free rates at one, two, three, and four years were 86.2%, 77%, 74%, and 65%, respectively.

**Conclusion:**

Cryoablation for lung nodules using 17-Gauge needles can achieve similar rates of survival and tumor control rates, similar or even lower complication rates as compared with other studies and multi-center trials using mixed sized needles.

## Background

For patients with a history of cancer who develop new lung nodules, surgical resection is frequently considered for both pathological evidence and therapeutic benefits [1–6]. Some individuals, however, may not be suitable surgical candidates due to personal medical conditions, such as inadequate pulmonary function or a history of repeated operations. In such cases, non-surgical alternatives, such as systemic drug therapy, radiotherapy, and percutaneous tumor ablation, are considered [3, 7–12]. Radiofrequency ablation (RFA), microwave ablation (MWA), and cryoablation (CA) are three of the most commonly used percutaneous tumor ablation techniques for lung nodules [12–21]. The capability of simultaneously employing multiple needles to sculpt the desired ablation zone and the ability to treat target tumors with minimal damage to vital structures are advantages shared by these techniques when image-guided. [13–15, 21–23] CA, in particular, is unique among the three methods because it produces visible ground glass opacity (GGO) due to frozen lung tissue (ice ball) in computed tomography (CT). This unique phenomenon has been utilized to estimate technical success and assess tumor treatment outcomes [17, 24, 25]. Furthermore, compared to thermal ablation methods, CA induces less pain during the procedure [2, 12, 17, 19, 22, 24–26]. Numerous large-scale studies and multi-center clinical trials [15, 24–28] have already examined the safety and viability of CA, revealing promising outcomes in terms of local tumor control and overall survival rates. In addition to examining the procedure’s complications, these studies [25, 27, 28] uncovered comparable complication rates across various ablation techniques for lung tumors. It is well known that complication rate of CT guided intervention is directly associated with needle size [29]. However, the potential impact of using different needle sizes (ranging from 13 to 17 gauge) in these trials has not been sufficiently addressed in terms of evaluating complications and treatment response [25, 27, 28, 30]. Therefore, the objective of this study is to assess whether the exclusive use of 17-gauge needles in cryoablation leads to different treatment responses, survival outcomes, and procedure-related complications for lung tumors.

## Materials and methods

### Study ethics

The Institutional Review Board (IRB) of our hospital granted approval for this investigation (approval number: 107,045). Patients who underwent cryoablation (CA) at our hospital between 2016 and 2020 were included in this retrospective study. An signed agreement was obtained from each patient which documented a thorough explanation of the limitations and potential risks associated with CA. The limitation are : CA is a local regional treatment and there is possibility of residual or recurrent tumor. The risks discussed encompassed pneumothorax, hemoptysis, pleural effusion, needle tract tumor seedings, procedure-related frostbite, infection, and fistula formation.

### Patients

Patients were referred for CA for management of lung nodules from chest surgeon, when not eligible for surgical treatment. Theare are certain conditions that surgery is not recommended such as insufficient pulmonary function, personal refusal by the patients, or the surgery is excluded by the guidelines. The process of shared decision making was well-documented in the medical records. Patients who were referred for this study had a medical history of confirmed primary cancers and presented with newly developed or growing lung nodules, as observed in three or more sequential computed tomography (CT) studies. Some patients had undergone prior surgical removal of pre-existing pulmonary nodules. The indications for referrals and the demographic information of the enrolled patients are summarized in Table 1.

**Table 1:** Baseline characteristics of patients (*N* = 41, 73 tumors treated)

Characteristics	Value
Age: Mean ± SD ( range ) years old	65.8 ± 8.39 (50–82 )
SEX M:F	21:20
Indications for referrals(n)	
Primary lung cancer post operation	20
Extrapulmonary malignancy with pathology proved lung metastases	15(8 colon,7 other location^a^)
Growing lung nodule with cancer history	6 (2 colon cancers,4 lung cancers)

^a^*1 renal cell carcinoma,1 urothelial cancer, 1 thymic cancer, 1 salivary gland cancer,1 prostate cancer, 1 pancreas cancer and one perineal leomyosarcoma*

The exclusion criteria were: ECOG performance status score equal to or more than 3, uncontrollable bleeding tendency with international normalization ratio (INR) more than 1.5. The patients who were not capable to hold his/her breath or hold still position. Evidence of progression of primary or metastatic tumor outside the lung; Patients with medical evidence of active infection.

### Cryoablation procedure

In this study, all cryoablation procedures were performed in the CT scanning room. Patients receiving antiplatelet therapies such as Aspirin or Clopidogrel (Plavix; Bristol-Meyers Squibb, New York, New York) were required to discontinue these medications 7 days prior to the cryoablation procedure. Local anesthesia was administered using 10-20mL of 2% Lidocaine injection. We utilized 17-gauge cryoablation needles (ICE ROD and ICEROD plus, Galil Medical, Yokneam, Israel) for the procedures. The positioning of patients varied between supine, prone, or decubitus positions to optimize the insertion route of the needles. CT scans were performed using a Philips brilliance 64 scanner (Philips Medical Systems, Netherlands). A non-contrast enhanced CT scan with a helical mode was initially conducted, with a 5 mm slice thickness and no gaps, at the start of the cryoablation procedure. This was followed by a 3 mm slice thickness after the CA needle was punctured through the skin, utilizing a tube voltage of 120Kv and a current of 89mA. The cryoablation procedure consisted of a freezing phase of 3 min, followed by a passive thawing phase of 3 min. This was then repeated with a freezing phase of 7 min, a passive thawing phase of 7 min, and finally a freezing phase of 10 min, followed by a 3-minute active thawing phase. CT studies of the entire lung were conducted prior to the cryoablation procedure (pre-CA CT), followed by CT studies of the ablation region after each thawing cycle to monitor the evolution of the iceballs. A post-procedure CT of the entire lung was performed after all the needles were removed. Our criterion for adequate cryoablation was to keep the tumors within a 5 mm margin of the minus 40 Celsius ablation zone, as specified in the cryoablation needle user manual published by the manufacturer (Galil Medical, Yokneam, Israel). The cryoablation procedure was performed by a board-certified radiologist with 20 years of experience in cross-sectional intervention (WCL).

### Safety and procedure related complication

Complications such as pneumothorax or hemoptysis were recorded by chart during and after the procedure. The criteria for pneumothorax is the presence of any newly found radiolucency between pleura and the lung parenchyma during and after CA procedure by CT. Hemoptysis was recorded on our Access database (Microsoft, WA, USA) when coughing with bloody sputum despite its amount was found during procedure. Any complaints by the patients during the CA procedure was also recorded on our Access database. Chart review was done for a period of 30 days following discharge to look for any documented discomfort or finding that may fall in the criteria by Common Terminology Criteria for Adverse Events (CTCAE)v3.0. The days of hospitalization was defined as the date of discharge minus the date the patient was admitted to ward.

### Follow up protocols and treatment response evaluation

**Fig. 1:**
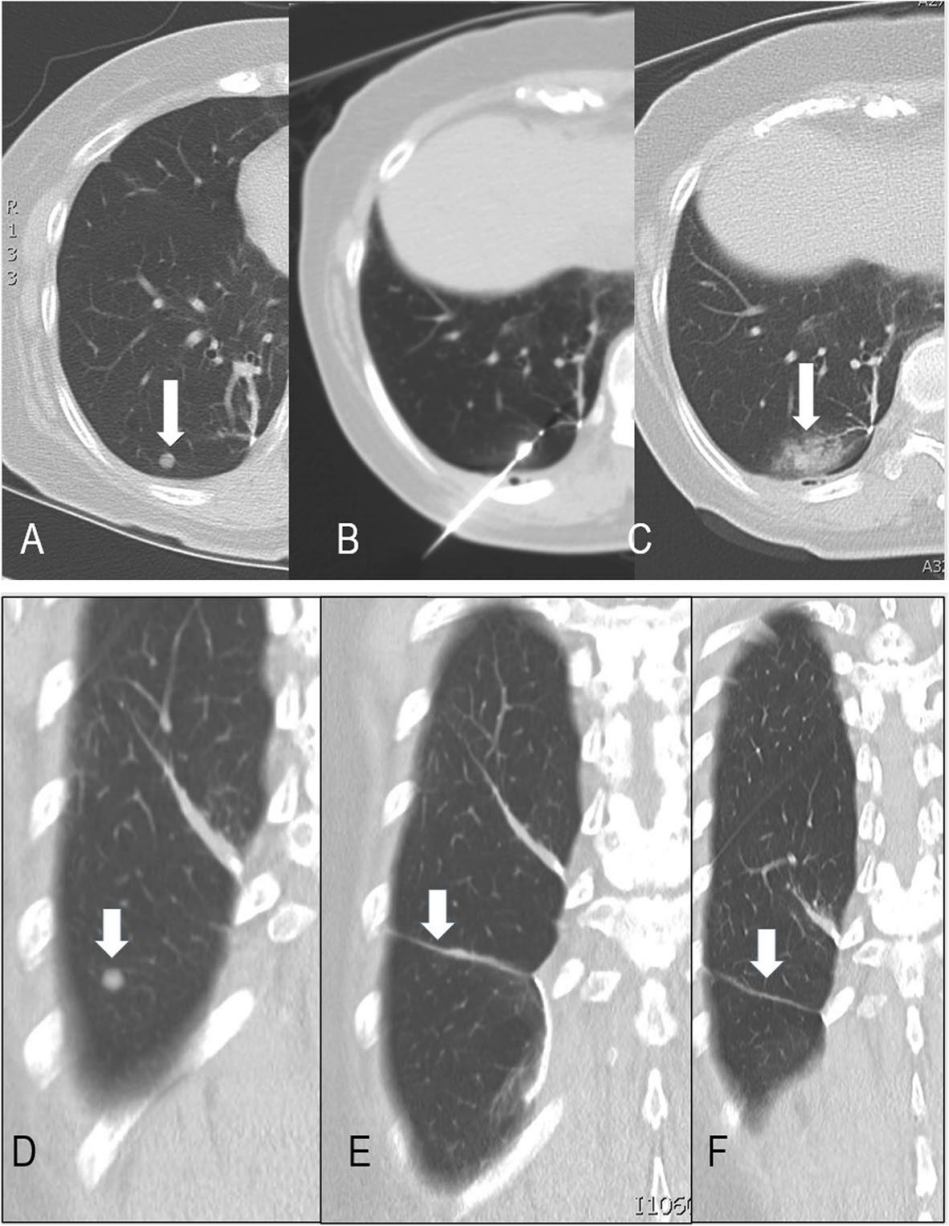
Complete response of nodule treated by CA procedure. A patient who was operated for renal cell carcinoma (RCC) and was confirmed with RCC metstasis at right lower lung (RLL) by opeartion. The patient developed a new 0.6 cm nodule at RLL.(**a**, marked by white arrow). The 17-Gauge cryoablation needle was punctured through the nodule, and the needle tip was placed at 2 cm distal to the nodule (**b**). CT image at axial and coronal planes obtained immediately at the end of the cryoabltion (CA) session showed complete coverage of the nodule by the ground glass opacity (**c**, marked by white arrow). Serial coronal CT images of the nodule at following time points: before cryoablation, 3 months post cryoablation and 3 years and 4 months after cryoablation (**d** and **f**), respectively. CT imge at **f** showed fine linear fibrosis which indicates complete response of the treated tumor

**Fig. 2:**
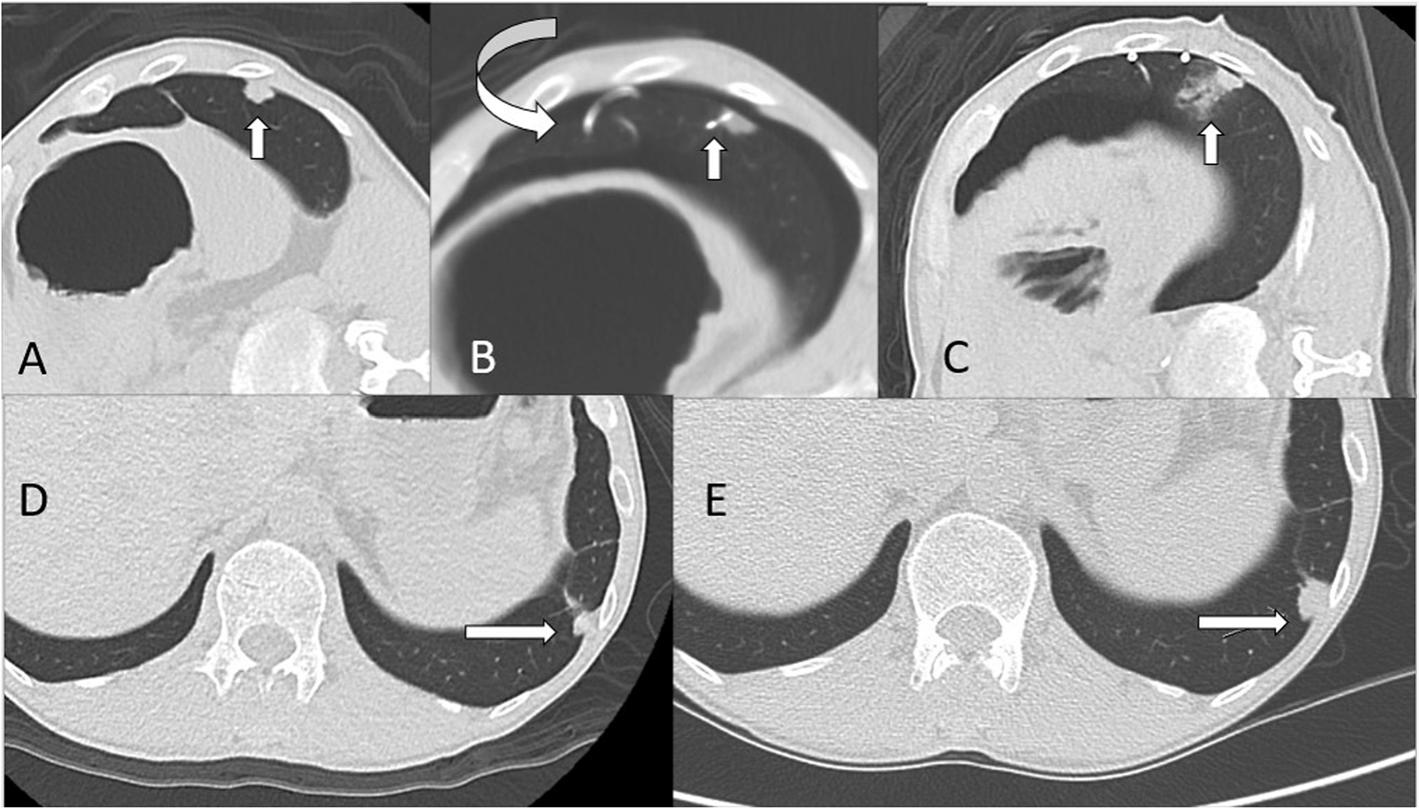
Local failure of nodule treated by CA procedure. A patient who was a victim of salivary gland cancer. This patient underwent surgery on the left lower lung (LLL) to remove a metastatic lesion, and subsequently developed a new nodule at LLL. CT images at axial plane shows the nodule before CA procedure (**a**, marked by white arrow). **b** shows the pigtail was inserted for drainage (curved arrow) accompanied by adjacent pneumothorax, and the cryoablation needle was punctured through the nodule (white arrow). The ground glass opacity represented outer border of the ablation zone is less than 0.5 cm at medial aspect of the target tumor(white arrow in **c**). The verdict of local failure was established after the comparison between the CT images at 3months and at 1year-4 month after CA procedure. (**d** and **e**, indicated by white arrows)

Follow up CT imaging after the CA procedure was done at three months interval. The 3-month post-cryoablation CT served as the baseline study for subsequent CT comparisons. This follow up protocol is in accordance with Taiwanese National Health Insurance Reimbursement Standards, which is also similar to the protocols mentioned in the ECLIPSE trial and in other articles [3, 8]. The scanning protocols were mentioned in the section of cryoablation procedure in Methods. The treatment response evaluation was performed using m-RECIST criteria for post ablated tumors, which was defined in detailed at SOLSTICE and ECLIPSE study [27, 28]. In brief, one should compare the largest diameter of the ablated tumor between the baseline study and the latest scan available. Complete response (CR)of index tumor(s) was defined as tumor ablation zone disappearance or reduction of at least 75%, partial response (PR) was defined as 30–75% decrease in size, stable disease(SD) was defined when there was less than 30% decrease and less than 20% increase in size, and local failure(LF) was designated as an increase of greater than 20% compared with the smallest diameter (nadir). Local tumor efficacy recorded as (CR), (PR), (SD) (LF) accordingly. The examples of the radiology evidence of complete response and local failures were shown in (Figs. 1 and 2). The treatment response was interpreted and recorded in the formal reports by four board certified radiologists with more than 10 years of thoracic CT imaging (WCL SHW PAL CYT).

### Statistics

#### Statistical analysis

We used SPSS software 26.0(SPSS, IL, USA) for statistical evaluation of outcome, patient demographics, and complication calculation. We have recorded continuous variables in mean, standard deviation (S.D.), minimal (MIN) and maximal value (MAX). The CA procedure time was defined as the time of first CT image was scanned to the time of procedure whole lung CT was scanned. The days of hospital stay were recorded as the interval between the patient was admitted and discharged, the days patient have spent in the hospital after the CA procedure were also calculated and recorded the way mentioned in SOLSTICE study [28].

Kaplan Meier method in the survival analysis function was chosen to calculate survival rates and tumor progression free rate at time points of 12 months, 24 months,36months and 48 months after CA procedure. In Kaplan Meier Methods, 95% confidence interval was chosen in the option list. The period of survival was calculated from the day of the first ablation procedure to time of death.

In the Kaplan Meier method, death by a direct or indirect consequence of lung cancer is designated as event as in the survival rate analysis and the patient who are still alive or loss of follow up were censored according to the chart review. The period of tumor progression free was calculated as the time from CA procedure to local failure. The tumor local failure was designated as event and loss of follow up was censored in the tumor progression free study, and the patients without progression were censored at the time of death or at the date of their last visit. The dates of the last OPD visit were updated until the end of 2020.

## Results

### Patients and CA procedures

Between Jan 2016 and July 2020, a total of 41 patients (21 males and 20 females) were recruited for CA procedure. Five patients with 6 targets were excluded from survival and local tumor control study, four patients were excluded as a consequence of their referral for tumor debulking, and the remaining two patients were lost in follow up study without base line post procedure CT. Out of the 41 patients, 9 did not undergo systemic therapy due to factors such as advanced age, personal choice, and impaired renal function. The treated nodules can be considered as metastases from primary lung cancers (total of 20 patients), or from malignancies other than lung (8 colon cancers, 1 renal cell carcinoma,1 urothelial cancer, 1 thymic cancer, 1 salivary gland cancer,1 prostate cancer, 1 pancreas cancer and one perineal leomyosarcoma) because these patients had previous pathology proved lung metastases. Also there were patients were treated with CA for they had prior cancer history with progressive growing lung nodule (2 colon cancers and 4 lung cancers). The characteristics of the patient involved were summarized in Table 1. A total of 62 procedures were performed for 73 tumors. We used a mean of 1.5 needles for each tumor, mean and range 1.5 ± 0.84(1–4) and an average of 1.16 tumor was treated at a single procedure. The mean tumor diameter is 1.48 cm. Mean days of hospital stays after the CA procedure is 2.27. Further details of CA procedure were documented in the Table 2.

**Table 2:** Details about cryoablation procedures (*N* = 41, 73 tumors treated)

Characteristics	Value
Number of tumors	73
Number of Procedures	62
Numbers of Needles Used Per Tumor Mean ± SD (range)	1.5 ± 0.84(1–4)
Number of Tumors Treated In One Procedure, Mean ± SD (range)	1.16 ± 0.37(1–2)
Number of Tumors Treated Per Patient, Mean ± SD (range)	1.49 ± 1.05 (1–6)
Mean Tumor Diameter ± SD ( range ) cm	1.48 ± 1.22(0.4–6.7^a^)
Mean Procedure Time ± SD (range), minutes	103.24 ± 38.3(58–232^b^)
Mean Days of Post Procedure Hospital Stay, days (range)	2.27(1–11)
Complication of CA Procedure	
Rate of Pneumothorax Per Procedure	51.6% (32/62)
Rate of Pigtail Drainage For Each Episode of Pneumothorax	21.8% (7/32)
Rate of Pigtail Drainage for pneumothorax per procedure	11.3% (7/62)
Rate of Hemoptysis Per Procedure	12.9% (8/62)

^a^for debulking purpose

^b^Second CA followed first CA in one procedure

### CA procedure related complications

Technical success was accomplished in 60/62(96.7%) procedures. Technical success was not achieved in two patients with two targets in each patient. Rapidly evolving pneumothorax during procedure was accountable for the failure to form the complete coverage of the target tumors in one patient. These four targets from the technical failures all contributed to local failure in the follow up.

For immediate and delayed complications of CA procedure, pneumothorax was found in 32 out of 62(52%) procedures while 7 out of 32 procedures require pigtail drainage. Hemoptysis was found in 8 out 62 procedures (12.9%), which were all self-limited and the hemoptysis ceased during or after procedure without further management. No delayed complication or discomfort such as fever, infection, local paresthesia, skin frostbite, fistula or focal hematomas was complained according to the chart review between the date one day after CA procedure and the date of his or her first OPD follow up which usually took place one or two weeks after discharge from ward (Table 2).

### The survival analysis and therapeutic effect evaluation

**Fig. 3:**
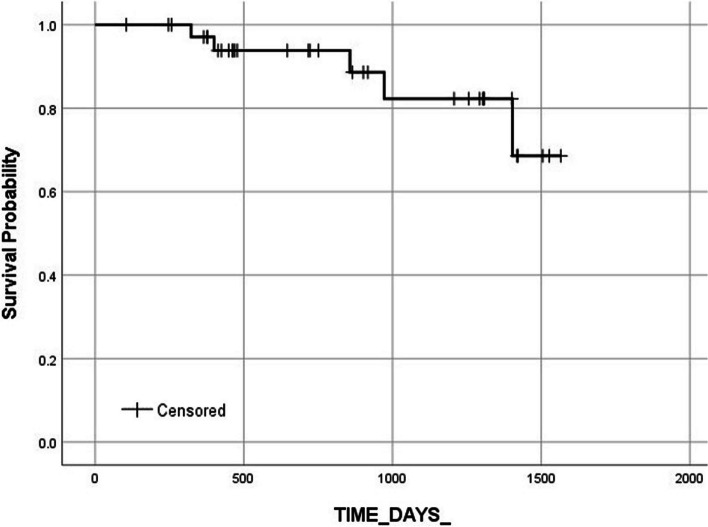
Overall survival of the patients after the percutaneous cryoablation of pulmonary nodules

In the study period, two patients have passed away in the first two years after their CA procedures. One of them passed away in the first year, the other one passed away in the second year. The survival curve using Kaplan Meier method was shown in (Fig. 3) which shows survival probability is 97% at first year and 94% at second year, 82% at third year and 67% at fourth year.

**Fig. 4:**
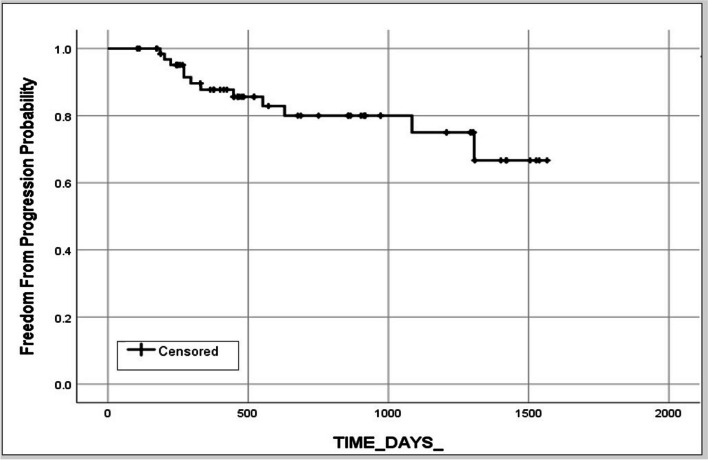
Time to progression for the treated nodules

According to the M-RECIST criteria, after five patients with six targets were excluded from the survival and tumor control study, there were 23 treated tumors showed complete response (CR),3 showed partial response (PR),27 showed stable disease (SD) 14 tumors were found to have local failure in 67 tumors, (Fig. 4). The local tumor progression-free response (local tumor efficacy) of the treated tumors was at 86.2% at 12 months and 77% at 24 months, 74% at 36 months and 65% at 48 months.

## Discussion

### Survival and tumor control rates

In this study we have shown the one- year, two-year, three-year, and four-year overall survival rate was 97%, 94%, 82% and 67%, respectively. These overall survival rates were comparable with the results reported in the multi-center clinical trials and other studies(97.6% at first year and 86.6% at second year by SOLSTICE study, 97.5% at first year and 84.3% at second year by ECLIPSE trial, and 89.4% at first year and around 85% by Moore, et al., respectively) [15, 19, 25–28]. Although the three year overall survival rate of 82% in our study showed slight higher than the similar studies (78% in Moore, et al. and 63% in ECLIPSE), the differences may be too small to be of statistical significance [19, 28]. These similar survival rates in different studies may indicate that CA procedure can yield similar survival despite primary or metastatic origin of the target lung nodules and various needle sizes (Table 3).

**Table 3:** Comparison of different studies reporting cryoablation for lung nodules

Studies^Ref no^	Moore,et al. [19]	ECLIPSE [26, 28]	SOLSTICE [27]	This Study	Leppel-mann, et al. [25]	Yashiro, et al. [15]	Inoue, et al. [22]
**Patient numbers**	45	40	128	41	10	71	117
**Origin of Targets: nodules = mixed origin**	lung	mets	mets	nodules	Head and neck mets	nodules	nodules
**Mean tumor size(cm)**	1.9	1.4	1.0 ± 0.6	1.48	1.6	1.28	1.4
**Follow up protocol: baseline/ months after baseline**	1 M/3 M	3 M/3 M	1 M/3 M	3 M/3 M	1 day or 1 M/3 M	1 M/3 M	1WK or 1 M/3 M
**Anesthesia Methods** **(LA local anesthesia** **GA General anesthesia)**	GA	GA67% conscious sedation 33%	GA 69% Conscious Sedation 29% LA 2%	LA	Concious Sedation/GA	Pentazzo-cine+ Atropine + LA	LA
**needle size(Gauge/G)**	13G/ 16G	17G/13G	17G /15G/ 13G	17G	16G	15G	13G/ 12G
**Pneumothorax per procedure %** **(N/A non applicable)**	51	N/A	N/A	52	38.2	N/A	61.7
**Pneumothorax require drainage per procedure% (N/A non applicable)**	12.8	18.8	26	11.3	14.3	N/A	17.6
**Hemoptysis per procedure % (N/A non applicable)**	40	N/A	N/A	12.9	33.3	N/A	36.8
**Overall Survival 1year%**	89.4	97.5	97.6	97	N/A	N/A	N/A
**Overall Survival 2year%**	Around > 85^a^	84.3	86.6	94	N/A	N/A	N/A
**Overall Survival 3 year%**	78.1	63.2	N/A	82	N/A	N/A	N/A
**Overall Survival 5 year%**	67.8	46.7	N/A	N/A	N/A	N/A	N/A
**Response Criteria**	Radiology &PET	M-RECIST	M-RECIST	M-RECIST	PET/CT and CT	Nadir+/- CE^b^	N/A
**Tumor progression free rate 1Y%**	N/A	94.2	85.1	86.2	88(estimated)	80.4	N/A
**Tumor progression free rate 2Y%**	N/A	N/A	77.2	77	65(estimated)	69.0	N/A
**Tumor progression free rate 3Y%**	N/A	83.3	N/A	74	N/A	67.7	N/A
**Tumor progression free rate 5Y%**	63.8^c^	75	N/A	N/A	N/A	N/A	N/A

^a^**Estimated from Fig. **1

^b^**CE = contrast enhancement**

^c^ **total at the end of follow up**

Secondly, in this study we have shown the tumor progression free rates were 86.2% at first year, 77% at second year. According to the statements mentioned in the studies by de Baere et al., direct comparison for local tumor control rate is difficult between different studies [25]. In this study, our tumor progression free rates at the first year was comparable with the numbers presented in these clinical trials (Table 3) [25, 27, 28]. The relatively larger mean tumor size (1.48 cm versus 1.4 cm in ECLIPSE study) may have contributed to the slightly lower local control rate found by our study. (86% versus 94% in ECLIPSE at first year) [27, 28]. In an article by Yashiro et al., which has reported the local control rate of 80.4% at first year and 69.0% at second year for lesions with mean size of 1.28 cm [15]. Further investigation regarding the likelihood of local failure in CA procedure when correlated with sizes of tumors may be of great clinical significance. There are reports discussing the correlation between cryoablation needle size and the resulting ablation zone, [31] these reports have not definitively elucidated the direct influence of needle size on tumor control efficacy. Our findings, which showed similar rates of survival and tumor control when compared with other studies and clinical trials, suggest that the data reported in these previous trials and studies may not have been significantly affected by variations in the sizes of cryoablation needles used.

In addition to the relatively larger sizes of our targets, the number of second year tumor progression free rate can be up to 83%, if the four targets which are not categorized as technical success were not included in the progression free period calculation. In the fact, the four targets which were not treated with technical success all ended up with local failures. Since the ECLIPSE study has reported 100% technical success rate and the SOLSTICE study has reported the 97.2% without further description of the outcome, more evidence may be required to clarify the relationship between technical success and the local tumor control in CA procedures [25, 27, 28].

### Complications

In this study, we observed a pneumothorax rate of 52% per procedure, which is consistent with findings from other studies summarized in Table 3, regardless of the needle size used for cryoablation [19, 22, 25–27]. However, the rate of pneumothorax requiring pigtail drainage in our study was 11.3% per procedure, which is among the lowest reported rates compared to other studies (17.6–25.9%) in Table 3 and the larger series cited [19, 22, 25–27]. Notably, the ECLIPSE 2021 study reported a much lower pneumothorax rate, with only 6.25% of procedures resulting in pneumothorax and 2% requiring drainage management. However, the details regarding the classification of pneumothorax were not provided in the ECLIPSE 2021 study, warranting further investigation for a comprehensive analysis. In a previous study by Inoue et al., it was proposed that patients who had undergone ipsilateral surgery and male patients were more predisposed to chest tube insertion during cryoablation. However, this observation was based on the use of 12 and 13-gauge needles and may not be applicable to recent and future studies utilizing smaller needle sizes, such as 17-gauge or less [22].

The second and final complication encountered in our cryoablation procedures was self-limited hemoptysis, which occurred in 12.9% of cases. This rate is lower than the reported rates of hemoptysis during and after cryoablation in previous studies, which ranged from 36.8 to 40%. This difference may be attributed to the use of a relatively smaller cryoablation needle (17-gauge) compared to the 12-16-gauge needles used in other studies [15, 19, 22, 26–28, 32]. Additionally, there are other reports that discuss complications associated with the use of 17 Gauge needles. Some of these reports have indicated a considerably higher hemoptysis rate (60%). It’s worth noting that this disparity in hemoptysis rates may be attributed to patient selection criteria, particularly focusing on centrally located tumors [33].

It is important to note the limitations of this study. It is a retrospective study with a single-arm design, and the number of enrolled patients is limited. The study population is heterogeneous, consisting of patients with different primary tumor origins and varying tumor sizes. The impact of systemic therapy before and after the cryoablation procedure was not taken into account, which may influence the evaluation of therapeutic effectiveness. Additionally, the study lacks long-term data on tumor control rates beyond five years, requiring further observation.

In conclusion, cryoablation using 17-gauge needles can achieve similar survival and tumor control rates, as well as comparable or lower complication rates, compared to multi-center trials that use various needle sizes.

## Data Availability

We promise to offer data supporting the results reported in the article upon request, please contact the corresponding author for the data.

## Declarations

We hereby confirm that all experimental protocols were approved by a named institutional and/or licensing committee. (IRB of Cathay General Hospital )

We confirmed the IRB of Cathay General Hospital waived the need for informed consent.
